# Social, ethical and behavioural aspects of COVID-19

**DOI:** 10.12688/wellcomeopenres.15813.2

**Published:** 2020-06-25

**Authors:** Wirichada Pan-ngum, Tassawan Poomchaichote, Giulia Cuman, Phee-Kheng Cheah, Naomi Waithira, Mavuto Mukaka, Bhensri Naemiratch, Natinee Kulpijit, Rita Chanviriyavuth, Supa-at Asarath, Supanat Ruangkajorn, Margherita Silan, Silvia Stoppa, Gianpiero Della Zuanna, Darlene Ongkili, Phaik Kin Cheah, Anne Osterrieder, Mira Schneiders, Constance R.S. Mackworth-Young, Phaik Yeong Cheah

**Affiliations:** 1 Mahidol Oxford Tropical Medicine Research Unit, Faculty of Tropical Medicine, Mahidol University, Bangkok, 10400, Thailand; 2 Department of Tropical Hygiene, Faculty of Tropical Medicine, Mahidol University, Bangkok, 10400, Thailand; 3 The SoNAR-Global Network, Mahidol University, Bangkok, 10400, Thailand; 4 Paediatric Ethics Committee; Research Ethics Committee, University Hospital of Padua, Padua, Italy; 5 Sabah Women & Children's Hospital, Ministry of Health, Malaysia, Kota Kinabalu, Sabah, Malaysia; 6 Centre for Tropical Medicine & Global Health, Nuffield Department of Medicine, University of Oxford, Oxford, UK; 7 Department of Statistical Sciences, University of Padua, Padua, Italy; 8 Luoghi di Prevenzione, Reggio Emilia, Italy; 9 Queen Elizabeth Hospital, Ministry of Health, Kota Kinabalu, Sabah, Malaysia; 10 Faculty of Arts & Social Science, Universiti Tunku Abdul Rahman, Kampar, Malaysia; 11 Department of Global Health and Development, London School of Hygiene and Tropical Medicine, London, UK

**Keywords:** COVID-19, social, ethics, qualitative, Thailand, Malaysia, United Kingdom, Italy

## Abstract

**Introduction**: Vaccines and drugs for the treatment and prevention of COVID-19 require robust evidence generated from clinical trials before they can be used. Decisions on how to apply non-pharmaceutical interventions such as quarantine, self-isolation, social distancing and travel restrictions should also be based on evidence. There are some experiential and mathematical modelling data for these interventions, but there is a lack of data on the social, ethical and behavioural aspects of these interventions in the literature.

Therefore, our study aims to produce evidence to inform (non-pharmaceutical) interventions such as communications, quarantine, self-isolation, social distancing, travel restrictions and other public health measures for the COVID-19 pandemic.

**Methods**: The study will be conducted in the United Kingdom, Italy, Malaysia, Slovenia and Thailand. We propose to conduct 600-1000 quantitative surveys and 25-35 qualitative interviews per country. Data collection will follow the following four themes: (1) Quarantine and self-isolation (2) social distancing and travel restrictions (3) wellbeing and mental health (4) information, misinformation and rumours. In light of limitations of travel and holding in-person meetings, we will primarily use online/remote methods for collecting data. Study participants will be adults who have provided informed consent from different demographic, socio-economic and risk groups.

**Discussion**: At the time of the inception of the study, United Kingdom, Italy, Malaysia, Slovenia and Thailand have initiated strict public health measures and varying degrees of “lockdowns” to curb the pandemic. These public health measures will change in the coming weeks and months depending on the number of cases of COVID-19 in the respective countries. The data generated from our study could inform these strategies in real time.

## Introduction

COVID-19 is a respiratory disease caused by a novel coronavirus (SARS-CoV-2) and causes substantial morbidity and mortality. At the time of the inception of the study, there are no vaccines to prevent COVID-19 infection with SARS-CoV-2 or therapeutic agents to treat COVID-19. Outbreak forecasting and mathematical modelling suggest that case numbers will continue to rise unless substantial public health measures are imposed ^1^.

Managing the COVID-19 pandemic poses a considerable challenge for global and public health actors. Responding institutions and organizations have to utilise non-pharmaceutical interventions such as quarantine, self-isolation, social distancing, travel restrictions and other public health measures ^2^.

In the current situation of expanding transmission and uncertainty, it is important to have evidence to inform such public health interventions to ensure maximum public acceptance, success, and minimum disruption to the lives of those affected. It is also necessary to understand how people acquire, interpret and act upon diverse information about COVID-19. This will help public health authorities with approaches to communication, and choice of communication channels and messaging.

Vaccines and drugs for the treatment and prevention of COVID-19 require robust evidence generated from clinical trials before they can be used. Decisions on how to apply non-pharmaceutical interventions such as quarantine, self-isolation, social distancing and travel restrictions should also be based on evidence. There are some experiential and mathematical modelling data for these interventions ^3, 4^, but there is a lack of data on the social, ethical and behavioural aspects of these interventions in the literature ^5, 6^.

Therefore, our study aims to produce evidence to inform (non-pharmaceutical) interventions such as communications, quarantine, self-isolation, social distancing, travel restrictions and other public health measures for the COVID-19 pandemic. We propose to conduct a mixture of quantitative surveys and qualitative interviews to answer our research questions.

## Description and rationale of study sites

At the time of the inception of the project, governments in Italy, UK, Slovenia, Malaysia and Thailand have initiated strict public health measures and varying degrees of “lockdowns” to curb the pandemic. We chose to include these countries in the study as they represent the worst and least affected countries in this pandemic as well as have various levels of stringency of government response.

Italy has recorded one of the highest number of COVID-19 cases in the world and has been restricting the movements of its residents since 9 March 2020. On 22 March, the Italian government ordered the shutdown of all non-necessary activities and movement between cities. In the United Kingdom, as of 23 March 2020, people have been requested to stay at home except to shop for basic necessities, attend to medical needs, travel to and from work (if they cannot work from home) and exercise once a day. On May 6 ^th^, the United Kingdom became the first country in Europe to pass 30,000 COVID-19 deaths. In Slovenia, strict preventive measures were taken early after the virus outbreak in Italy, a neighbouring country of Slovenia. The epidemic was declared on 12 ^th^ March, followed by complete lockdown of all non-essential activities. Movement was only allowed within individual municipalities.

The Malaysian government declared a “Movement Control Order” on 16 March 2020 which prohibits mass movements and gathering, closure of non-essential businesses and closing its borders. On 25 March 2020, the Thai Prime Minister declared a state of emergency in Thailand.

These public health measures will change in the coming weeks and months depending on the number of cases of COVID-19 in the respective countries. The data generated from our study could inform these strategies in real time.

## Master protocol

### Overarching objective

To produce evidence to inform (non-pharmaceutical) interventions such as communications, quarantine, self-isolation, travel restrictions and other public health measures for the COVID-19 epidemic.

### Specific objectives

1. To understand the factors that impede and facilitate the compliance of quarantine, self-isolation, social distancing and travel restrictions at different phases of the epidemic;

2. To explore people’s understanding about quarantine, self-isolation, social distancing and travel restrictions;

3. To identify information sources and investigate any rumours and misinformation

### Research questions

1. What are the perceptions and experiences regarding quarantine, self-isolation, social distancing, and travel restrictions?

2. What are the economic, social and ethical impacts (e.g. lost wages, challenges in child care, food and household supplies, loneliness) of quarantine, self-isolation, social distancing, and travel restrictions?

3. How do people understand and define these terms (quarantine, self-isolation, social distancing and travel restrictions)? What are the barriers and enablers for complying with these measures? How do people cope?

4. What are individuals from various communities (e.g. parents, caretakers, people from different occupations) most fearful of (e.g. loss of wages, dying, spreading to others; being unable to care for children/older people/other family members; self-isolation; being unable to get care)?

5. What are the rumours and misinformation circulating in social media, conversations and discussions? To what extent has public information been clear? How do people obtain information?

### Project timeline

The duration of data collection is three months. It is expected that the primary analyses will be completed by December 2020. The secondary, more in-depth analyses will take another year.

### Study design

We will conduct a mixed methods study using quantitative surveys and qualitative interviews to obtain contextual information from communities on the following four themes:

(1) Quarantine and self-isolation

(2) Social distancing and travel restrictions

(3) Wellbeing and mental health

(4) Information, misinformation and rumours

### Study sites

The study will be conducted in the UK, Italy, Slovenia (quantitative survey only), Malaysia and Thailand. In light of limitations of travel and holding in-person meetings, we will primarily use online/remote methods for collecting data. In person qualitative interviews may be conducted when it is safe to do so and in compliance with local regulations.

### Study participants

The study participant will be adults who have provided informed consent.

Inclusion criteria:

- Adults (age may vary by country)
- Residing in Thailand, Italy, Slovenia, Malaysia or the UK
- Provided consent to participate in the study
- Able to use a computer or smart phone

Exclusion Criterion:

- Individuals who are illiterate (because the data collection is online and the survey is self- administered)

**Note:** Individuals who are tested positive for coronavirus will not be excluded unless they meet the exclusion criterion

### Study procedures

Recruitment:

Invitation to join the survey will be sent through professional and personal contacts via email, other online media, and recruitment posters.

There will be three ways of recruiting people to participate in the qualitative interviews:

1) Via quantitative survey – at the end of the online survey, participants will be asked if they wish to take part in the qualitative research. They will be asked to click on a link that will take them to a different webpage that will enable them to provide their email address if they are interested to participant in the qualitative interview. The email address will not be linked to the survey answers. The study team will email the people who provided their email address to seek invite them to join the qualitative interview.

2) Via recruitment posters advertised on partner websites and social media. Interested participants can contact the study team directly. The study team will email the people who provided their email address to join the qualitative interview.

3) Snowball sampling and referrals will be used to reach additional participants and those who are not familiar with emails or do not have internet access, in order to facilitate greater diversity of participants in the sample. The steps include:

- Recruitment via personal and professional networks, including families, friends and colleagues who may represent or have contact with persons we wish to include in order to gain maximum variation within our sample. 

- The member of the research team making contact will give information on the research project, including an invitation for interested individuals to take part in the study.

- The study team will then contact the potential participants, provide further information about the study and set up an interview.

Participants in the qualitative study will be selected with the aim to recruit a maximum variation sample, based on characteristics including participant age, gender, risk and socio-economic status.

Informed consent:

Consent will be taken separately for participation in the quantitative survey and the qualitative interviews. Participants will be asked to provide consent online for the quantitative survey, and either or in-person or online for the qualitative study.

### Data collection methods, study participants, sample size and topics for discussion

We will conduct the following:

Quantitative method: About 600 to 1000 online surveys will be obtained per country. No formal sample size calculation has been performed. This number is more than what is recommended for a mixed methods study ^7^. Furthermore, it is feasible for data collection within three months. Surveys will be self-administered. The online survey will be available in English, Thai, Slovenian and Italian (see extended data ^8^).

Qualitative method: Online (via MS Teams, telephone, or other approved platforms) interviews and focus group discussions with 25–35 participants will be conducted per country. Actual numbers will depend on context, changes in epidemic, and data saturation. Qualitative data collection will be conducted by in-country interviewers in the language (English, Malay, Thai or Italian) preferred by the participant. A pre-prepared topic guide will be used to direct conversations (extended data ^9^).

Questions for both quantitative and qualitative will be guided by the following themes:

(1) Quarantine and self-isolation

(2) Social distancing and travel restrictions

(3) Wellbeing and mental health

(4) Information, misinformation and rumours

We will target different communities (based on age, gender, risk and socio-economic status) within Thailand, Italy, Malaysia and the UK. Potential communities include those working in the healthcare sector, tourism industry, taxi drivers, market vendors, university students and public advisory groups.

### Data analysis and management

Quantitative data: Quantitative survey data will be entered and analysed using SPSS software. Data may also be analysed using Stata 15.0 (or later) software. The quantitative data will be retrieved from the online survey platform. The data can be accessed real time to monitor the response rate to boost the sharing of the link to reach the target sample size. Once the data have been collected we will review the data and bring together the related responses. Frequency counts and percentages will be used to summarise categorical data. Associations between categorical variables will be assessed using the Pearson Chi-Square tests or Fisher’s exact tests as appropriate. Data will be presented in different tables, graphical displays and summary statistics. Further analysis to investigate the relationship between different variables will be performed. Tests of significance will be performed at 5% significance level.

Qualitative data: Interviews and focus group discussions will be audio recorded where possible ^10^. They will be transcribed, cleaned and translated to English where necessary and appropriate, and exported into NVivo (© QSR International Pty Ltd) or equivalent software that will be used to manage the data. In all other cases, detailed written notes taken immediately after each interview will serve as the data for analysis ^10^. Audio files will be kept in country until they have been transcribed and interview transcripts will be kept securely. All audio files will be destroyed once all transcripts have been completed and verified. Codes will be established for each participant to enable appropriate collation of data sets, themes and sub-themes for qualitative analysis. Qualitative data analysis will be based on thematic content analysis. Initial themes and categories will be developed iteratively through successive coding of the raw data transcripts, and informed by the research objectives, issues emerging from the raw data and media. To support the validity and trustworthiness of data analysis, two researchers will independently develop their own coding categories, followed by a discussion of similarities and differences. Where information gathered by different methodologies is contradictory rather than complementary, divergences will be outlined and discussed in reports and publications.

Qualitative and quantitative data will be analysed by country and pooled for comparison between countries.

Direct access will be granted to authorised representatives from the sponsor, ethics committees, and regulatory authorities to ensure compliance with regulations.

De-identified data will be stored digitally and indefinitely. De-identified data may also be shared with other research groups. All applications for data sharing will be reviewed by the MORU Data Access Committee in consultation with country Principal Investigators. All researchers accessing the data need to adhere to a set of terms and conditions that aim to protect the interests of research participants and other relevant stakeholders.

Data generated from this study will adhere to the 2016 “Statement on data sharing in public health emergencies”.

### Quality control and quality assurance procedures

The study will adhere to the relevant guidelines for surveys and qualitative research. All interviewers, and transcribers will be trained prior to the study.

The study will be conducted in accordance with relevant national and international guidance and regulations.

Survey questions have been pilot-tested and have undergone pilot testing in accordance with established cognitive interviewing and questionnaire design methodology ^11^.

### Discontinuation/withdrawal of participants from study

Each participant has the right to withdraw from the study at any time. Withdrawal of consent to participate from this study will result in exclusion of the data for that participant from analysis. Withdrawn participants will not be replaced. If identified, the reason for withdrawal will be recorded.

In addition, investigators may discontinue participation of any individual from this study at any time if the investigator considers it necessary for any reason.

Audio recordings will be deleted if a participant decides to withdraw mid-interview. For those participating in a focus group discussion, if a participant withdraws mid-focus group, those portions of the audio recording that capture their views will be deleted.

### Ethical and regulatory considerations

Risk and harm:

This is a minimal risk study posing minimal risk and harm to the participants.

The main ethical issues in this study relate to privacy and confidentiality. Care will be taken to maintain privacy during the audio recording of interviews and interactions with individual participants.

Risk of exclusion:

Due to the fact that the survey is online, there is a risk that people who are illiterate, people in rural communities and others who do not have internet access may not be reached.

Approvals:

The protocol, informed consent form, participant information sheet and any associated materials have been approved by the following ethics committees:

Oxford Tropical Research Ethics Committee (OxTREC, 520-20); University Tunku Abdul Rahman Scientific and Ethical Review Committee (U/SERC/63/2020); Ministry of Health Malaysia Medical Research and Ethics Committee (NMRR-20-595-54437-IIR), Republic of Slovenia National Ethics Committee (NMEC 120-237/2020/7) and the Ethics Committee of the Faculty of Tropical Medicine, Mahidol University (MUTM 2020-031-01). Ethics committee approval is not required for the study to be conducted in Italy as OxTREC covers it.

The Chief and country Principal Investigators will submit and, where necessary, obtain approval from the above parties for all amendments to the original approved documents.

Participant confidentiality:

The study team members will ensure that the participants’ confidentiality is maintained. The participants will be identified only by a participant identification number on all study documents and any electronic database. All documents will be stored securely and only accessible by study staff and authorised personnel. The study will comply with the EU General Data Protection Regulation (GDPR) and country specific data protection regulations.

Compensation:

Participants will not be offered any payment to complete the online survey. Participants who take part in the interviews and focus groups will be compensated in cash or in kind according to country guidelines (e.g. shopping vouchers) for their time.

Benefits:

There will be no immediate benefits for any of the study participants. The chief benefit to participation in this study is that participants will be afforded the opportunity to contribute to the generation of new knowledge.

Public engagement and involvement:

As part of the development of our study and data collection tools, we have conducted a series of public engagement or public involvement activities e.g. with the existing community advisory boards ^12, 13^ and Bangkok Health Research Interest Group. We believe that public engagement and involvement is necessary for ethical research ^14^. The INVOLVE group, the UK’s national advisory group for public involvement defines public involvement as research that is actively carried out ‘with’ or ‘by’ members of the public rather than ‘to’, ‘about’, or ‘for’ them.

Reporting:

The country Principal Investigators shall submit progress reports and end-of-study reports to the relevant ethics committees.

### Publication policy

The Chief Investigator will lead writing and reviewing of drafts of the manuscripts, abstracts and any other publications arising from the overall study. The country Principal Investigators will lead the writing of country publications in collaboration with the Chief Investigator. Authorship will be based on the set of criteria outlined by the International Committee of Medical Journal Ethics. The study results will also be published as regular short reports, and an evaluation report of the online data collection approach.

### Dissemination of information

Regular short reports will be made available in real time to public health authorities and researchers, including:

- WHO COVID-19 Research Roadmap Social Science Research Working Group (the Chief Investigator, PYC is a member of the group)
- UK Emergency Preparedness and Response Health Protection Research Unit
- Health professionals and healthcare staff from the Department of Disease Control, Thailand Ministry of Public Health, e.g. Division of Communicable Diseases, Bureau of Epidemiology, Health Intervention and Technology Assessment Program (HITAP)
- Italian Ministry of Health
- Italian Ministry of Innovation Technologies and Digitalisation
- Ministry of Health, Malaysia
- Network of research ethics committees in participating countries
- Research networks for pandemics and infectious diseases e.g. SoNAR-Global Network, Public Health Emergency Preparedness and Response Ethics Network (PHEPREN)
- University researchers and other organisations working on COVID-19 response

Results of the will be published as academic publications and presented at academic conferences. They will also be available in lay language for dissemination to the wider public.

Details of the study are available from the study website: https://www.tropmedres.ac/covid-19/sebcov

### Study status

Ethics approvals have been obtained. Data collection is ongoing.

## Data availability

### Underlying data

No data are associated with this article

### Extended data

Zenodo: Online survey questions: Social, ethical and behavioural aspects of COVID-19. http://doi.org/10.5281/zenodo.3764913 ^8^

This project contain the following extended data:

- SEBCOV_Survey_AllVersions_V1.0_24Apr2020.pdf (Study survey questions in English, Thai and Italian)

Zenodo: Topic guide for qualitative study: Social, ethical and behavioural aspects of COVID-19. http://doi.org/10.5281/zenodo.3777934 ^9^

This project contains the following extended data:

- SEBCOVTopicGuide_30April2020.pdf (Topic guide for qualitative study)

Data are available under the terms of the Creative Commons Attribution 4.0 International license (CC-BY 4.0).
