# Supplementary material for: Social, ethical and behavioural aspects of COVID-19
Source: Wellcome Open Res. 2020 Jun 25;5:90. Originally published 2020 May 7. [Version 2] doi: 10.12688/wellcomeopenres.15813.2 (PMC7355217; doi:10.12688/wellcomeopenres.15813.2)
Supplement: Peer review history

# Peer review: Reviewer response for version 2

**DOI:** 10.21956/wellcomeopenres.17666.r39238

**Published:** 2020-07-10

**Authors:** Teck Chuan Voo

**Affiliations:** 1 Centre for Biomedical Ethics, Yong Loo Lin School of Medicine, National University of Singapore, Singapore, Singapore

No further comments.

Is the study design appropriate for the research question?

Partly

Is the rationale for, and objectives of, the study clearly described?

Partly

Are sufficient details of the methods provided to allow replication by others?

Partly

Are the datasets clearly presented in a useable and accessible format?

Not applicable

Reviewer Expertise:

Research ethics & public health ethics, and their intersection

I confirm that I have read this submission and believe that I have an appropriate level of expertise to confirm that it is of an acceptable scientific standard.

# Peer review: Reviewer response for version 1

**DOI:** 10.21956/wellcomeopenres.17341.r38676

**Published:** 2020-06-01

**Authors:** Sharon Kaur Gurmukh Singh

**Affiliations:** 1 Faculty of Law, University of Malaya, Kuala Lumpur, Malaysia

This is an important study given that in the absence of any vaccine or effective treatment of COVID-19, State actors will have to rely on non-pharmaceutical interventions such as quarantine, self-isolation, social distancing and travel restrictions. It is vital that such interventions are based on evidence and because these measures are social in nature, it is appropriate and necessary to conduct the study using the proposed methods.

 Significantly, the success of public health interventions are to a large extent dependent on public trust and the willingness of the public to adhere to the measures set out by the State. Transparency and credible state actions are essential criteria of establishing and maintaining public trust. State actors may design interventions and implement them in ways that they believe will meet these criteria, but until and unless there is evidence to demonstrate how the interventions are received and what the public are thinking, it is difficult to ascertain the genuine effectiveness of these strategies. The objectives of the study and research questions articulated by the authors will go some way toward answering a number of these key issues. 

 However, it is important to recognise that how the public experience and understand non-pharmaceutical public health interventions will be heavily influenced by contextual features such as  political, social, cultural and religious norms. Therefore, any analysis must recognise the possible limitations of the evidence. Also, and perhaps more significantly, given that the method of data collection is online, there is some risk that people in rural communities or marginalised populations may not be reached. There is growing evidence that marginalised populations at put at greater risks by these interventions. A number of international organisation have highlighted the increased risk faced by migrants and stateless persons  in Malaysia. It is also important to  hear the voices of key populations at risk, their experiences and concerns.

Is the study design appropriate for the research question?

Yes

Is the rationale for, and objectives of, the study clearly described?

Yes

Are sufficient details of the methods provided to allow replication by others?

Yes

Are the datasets clearly presented in a useable and accessible format?

Yes

Reviewer Expertise:

Research Ethics

I confirm that I have read this submission and believe that I have an appropriate level of expertise to confirm that it is of an acceptable scientific standard.

## References

[1] : Public health and ethical considerations in planning for quarantine. *Yale J Biol Med*.2005;78(5) :329-34 17132339PMC2259156

[2] : Public health strategies for pandemic influenza: ethics and the law. *JAMA*.2006;295(14) : 10.1001/jama.295.14.1700 1700-4 10.1001/jama.295.14.1700 16609092

[3] : Pandemic Influenza Risk Management.2017; Reference source

[4] : Protecting public trust in immunization. *Pediatrics*.2008;122(1) : 10.1542/peds.2008-0987 149-53 10.1542/peds.2008-0987 18595998

[5] : Public health response to influenza A(H1N1) as an opportunity to build public trust. *JAMA*.2010;303(3) : 10.1001/jama.2009.2023 271-2 10.1001/jama.2009.2023 20085957

# Response

**Published:** 2020-06-23

**Authors:** Phaik Yeong Cheah

**Affiliations:** Mahidol Oxford Tropical Medicine Research Unit, Thailand

Many thanks for your review and valuable comments.

# Peer review: Reviewer response for version 1

**DOI:** 10.21956/wellcomeopenres.17341.r38677

**Published:** 2020-05-28

**Authors:** Teck Chuan Voo

**Affiliations:** 1 Centre for Biomedical Ethics, Yong Loo Lin School of Medicine, National University of Singapore, Singapore, Singapore

This paper describes a protocol to collect empirical evidence related to the social, behavioural and ethical aspects of non-pharmaceutical interventions (NPI) implemented in response to the COVID-19 pandemic. The protocol is reasonably described, and the data collected could be useful for informing NPIs, though what this means could be given more thought and clarity. The authors appear to justify this study by analogizing NPI to the pharmaceutical interventions (PI) - just as we need evidence for PI before we use them, we need evidence for NPI. However, the evaluative frame for PI is clear- are they safe and effective? NPI could be evaluated in terms of compliance, and the proposed study's data could be used to understand factors that support or undermine compliance. The data it would collect seems less useful to inform ethical evaluation of NPIs, which depends on public health ethical principles such as least infringement, and harm minimization or mitigation. So some research questions could be introduced to understand for e.g. whether there were any government measures to mitigate the burdens of NPIs and whether these are sufficient from the perspective of the participants, types of individual coping strategies etc. 

 In addition, the study's inclusion criteria - able to use computer/smart phone - might exclude members of communities who lack access to these resources and who are critical to NPI efficacy in limiting spread. Understandably, the study has to rely on online modes of data collection in the current situation. I'm not sure if the study design should explicitly acknowledge this limitation or whether this could simply be stated in any subsequent publication. 

 Other comments:

1. It is not clear why the study chooses to focus on the 4 countries – some explanation would be helpful.
2. "At the time of writing, there are no... therapeutic agents to treat COVID-19." This needs some updating on the efficacy of Remdesivir in shortening recovery time if administered early.
3. "Outbreak forecasting and mathematical modelling suggest that case numbers will continue to rise." What are the assumptions or under what conditions would this happen?
4. “To identify information sources and investigate any rumours/misinformation and to reverse and correct any rumours/misinformation about COVID-19” – I don’t think that the underlined portion – which is an intervention – is or should be part of the study, unless you mean to identify when and how misinformation has been reversed and corrected.
5. “What are the rumours circulating in social media, conversations and discussions?” – this is one of the research questions but the objective is to examine “rumours/misinformation”. Rumours may or may not be misinformation so the research question can be revised to be consistent with the objective. Is there a need to examine when rumours turn out not to be misinformation?

Is the study design appropriate for the research question?

Partly

Is the rationale for, and objectives of, the study clearly described?

Partly

Are sufficient details of the methods provided to allow replication by others?

Partly

Are the datasets clearly presented in a useable and accessible format?

Not applicable

Reviewer Expertise:

Research ethics & public health ethics, and their intersection

I confirm that I have read this submission and believe that I have an appropriate level of expertise to confirm that it is of an acceptable scientific standard, however I have significant reservations, as outlined above.

# Response

**Published:** 2020-06-23

**Authors:** Phaik Yeong Cheah

**Affiliations:** Mahidol Oxford Tropical Medicine Research Unit, Thailand

Thank you Teck Chuan for your valuable comments. I have updated the various sections accordingly. As for the update on treatment of COVID, I did not mention remdesivir, but changed the sentence so that this protocol will remain current and does not need updating every time a new drug/intervention shows promise.
